# Dietary Temperature’s Influence on Energy Balance in Humans: Protocol for a Randomized Controlled Trial and Crossover Design

**DOI:** 10.2196/42846

**Published:** 2023-03-03

**Authors:** Reza Rastmanesh, Kyle D Flack

**Affiliations:** 1 American Physical Society Maryland, MD United States; 2 Department of Dietetics and Human Nutrition University of Kentucky Lexington, KY United States

**Keywords:** dietary temperature, energy intake, energy conservation, heat shock proteins, clinical trial, design of study

## Abstract

**Background:**

According to the first law of thermodynamics, energy cannot be created or destroyed in an isolated system. Water has a characteristically high heat capacity, indicating that the temperature of ingested fluids and meals could contribute to energy homeostasis. Citing the underlying molecular mechanisms, we present a novel hypothesis that states that the temperature of one’s food and drink contributes to energy balance and plays a role in the development of obesity. We provide strong associations with certain molecular mechanisms that are activated by heat and correlate them with obesity and a hypothetical trial that could test this hypothesis. We conclude that if meal or drink temperature proves to contribute to energy homeostasis, then depending on its contribution and scale, future clinical trials should attempt to adjust this effect when analyzing data. In addition, previous research and established relationships of disease states with dietary patterns, energy intake, and food component intakes should be revisited. We understand the common assumption that thermal energy in food is absorbed by the body during digestion and dissipated as heat into the environment, not contributing to the energy balance. We challenge this assumption herein, including a proposed study design that would test our hypothesis.

**Objective:**

This paper hypothesizes that the temperature of ingested foods or fluids influences energy homeostasis through the expression of heat shock proteins (HSPs), especially HSP-70 and HSP-90, which are expressed to a greater extent in obesity and are known to cause deficits in glucose metabolism.

**Methods:**

We provide preliminary evidence supporting our hypothesis that greater dietary temperatures disproportionally induce activation of both intracellular and extracellular HSPs and that these HSPs influence energy balance and contribute to obesity.

**Results:**

This trial protocol has not been initiated and funding has not been sought at the time of this publication.

**Conclusions:**

To date, no clinical trials are available regarding the potential effects of meal and fluid temperature on weight status or its confounding effects in data analysis. A potential mechanism is proposed as a basis by which higher temperatures of foods and beverages might influence energy balance via HSP expression. On the basis of the evidence supporting our hypothesis, we propose a clinical trial that will further elucidate these mechanisms.

**International Registered Report Identifier (IRRID):**

PRR1-10.2196/42846

## Introduction

### Background

The first law of thermodynamics, also known as the law of conservation of energy, states that energy can neither be created nor destroyed in an isolated system [1]. The human body has its own mechanisms to adapt to changing temperatures; however, it is possible that higher or lower temperatures of food and beverages (referred to as dietary temperature) impose some implications on the energy demands of the body. On the basis of diet composition, chewing habits, and the amount of fluid (and its corresponding temperature) ingested, the human body is exposed to a wide range of temperatures [2,3]. This exposure affects the entire gastrointestinal tract, being greatest at the mouth, followed by the esophagus and stomach, with the latter exposed for a greater time [4-7]. Indeed, there have been several studies relating dietary temperature to gastric function [6], frequency of gastric myoelectrical activity [7], gastric emptying time, gastrin release, gastric acid secretion [8], esophageal cancer [9,10], and intraluminal upper gastrointestinal temperature and motility [5]. Although when it comes to energy metabolism, it has been assumed that higher dietary temperatures are dissipated during digestion and do not contribute to energy balance.

One of the most immediate responses of body cells to higher temperatures is the induction and increased synthesis of heat shock proteins (HSPs), especially HSP-70 [11,12] and HSP-90 [13,14]. HSP-70 [15] and HSP-90 [16] possess N-terminal adenosine triphosphatase fragments at their ends and tightly regulate adenosine triphosphate (ATP) activity and ATP hydrolysis [13,17-19], providing the heat energy to drive the adenosine diphosphate to ATP conversion. Indeed, cells exposed to higher temperatures increase HSP activity [11-14], and the time profile of peripheral blood mononuclear leukocytes HSP-70 response to in vitro heat shock is temperature dependent [11,20]. For instance, under in vitro hyperthermic conditions (40-41 °C), the time course was characterized by a sharp rise in HSP-70 concentration immediately after heat shock treatment (*P*<.05 for 40 °C at 0 hours), followed by a steady and progressive decline over time [11].

**Figure 1:**
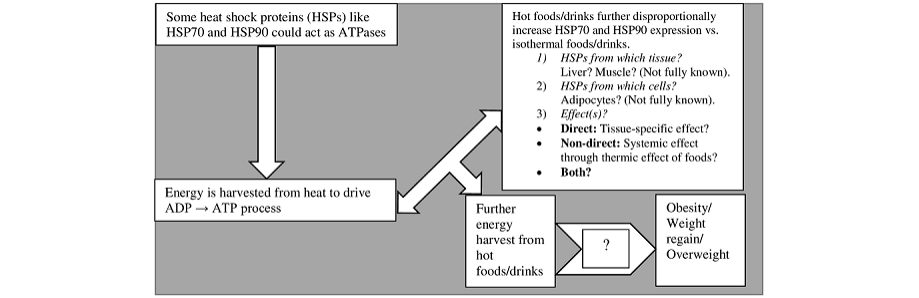
Hypothetic mechanism by which hot meals or drinks may contribute to energy balance. ADP: adenosine diphosphate; ATP: adenosine triphosphate; ATPase: adenosine triphosphatase.

We have already shown that HSPs can extract energy from the environment (here, dietary temperatures), thereby contributing to the energy balance. The mathematical calculations are beyond the scope of this paper, and interested readers may read our previous paper [21]. The summary is graphically illustrated in Figure 1.

Importantly, obesity has been linked to HSP expression, specifically for HSP antibodies (HSP-27 [22], HSP-72 [23], HSP-60, HSP-65, and HSP-70 [24,25]). The table in the Multimedia Appendix 1 [22,24-26] lists the studies investigating the relationship between anti-HSP antibodies and indexes of body mass and body composition. Specifically, serum HSP-27, HSP-60, HSP-65, HSP-70, and HSP-72 antibody levels are significantly increased in people with obesity compared to people with a normal weight [22-26]. Furthermore, in the diet-induced obese mouse model of insulin resistance, HSP-90 inhibitors activate the heat shock factor 1 stress response pathway and improve glucose regulation [27]. A similar experiment on diet-induced obese mice supports this hypothesis in which HSP-90β knockdown reverses insulin resistance and improves glucose tolerance [28].

### Evidence Supporting Our Hypothesis

#### Intracellular Versus Extracellular HSPs

HSPs help to maintain cellular homeostasis through a mechanism called thermotolerance. Cells exposed to mild stress induce HSPs, which later protect them against subsequent stress. In cells exposed to severe stress, HSPs promote apoptosis. HSPs meanwhile exist in extracellular fluids and contribute to immunomodulation [29]. It is noteworthy that the intracellular HSP function may be different from that of extracellular HSPs, as the latter appears to manifest a function different from the well-known chaperone role [29]. For instance, intracellular HSP-70 exerts a profound anti-inflammatory effect, whereas extracellular HSP-70 activates proinflammatory pathways [30]. In the case of obesity, both intracellular [31] and extracellular [32] HSP-70 levels are significantly greater in participants with obesity than in lean controls. It has been proposed that the ratio of extracellular medium HSP-70 to intracellular HSP-70 contents may be a determining factor to trigger a chronic proinflammatory status, which leads to insulin resistance and the development of type 2 diabetes mellitus (T2DM) [30].

#### Weight Regain and HSPs

In the only published randomized controlled trial (RCT) linking weight regain to HSPs, Roumans et al [33] explored the expression of stress proteins during weight loss and weight maintenance concerning weight regain. They compared the in vivo findings with the results from in vitro cultured human Simpson-Golabi-Behmel syndrome (SGBS) adipocytes. In total, 18 healthy participants participated in an 8-week dietary program with a 10-month follow-up period. They categorized the participants as weight maintainers or weight regainers based on their weight changes during the intervention. Abdominal subcutaneous adipose tissue biopsies were conducted before and after the diet and after the follow-up. In vitro–differentiated SGBS adipocytes were starved for 96 hours with low (0.55 mmol/L) glucose. Weight regainers showed increased expressions of calnexin, β-actin, HSP-27, HSP-60, and HSP-70. Changes in HSP-27 and HSP-70 and β-actin levels were linked to HSP-60, a novel key factor in weight regain after weight loss. SGBS adipocytes manifested increased concentrations of β-actin and HSP-60 after 96 hours of glucose restriction [33]. In another RCT, 20 young overweight men without metabolic syndrome participated in a 3-week residential program on a low-fat diet and moderate aerobic exercise. After 3 weeks of the diet program, significant reductions in BMI, serum lipids and lipid ratios, and oxidative markers were recorded (*P*<.05), along with the reduced expression of HSP-90 and HSP-27 [34].

There is ample literature on the effects of heat stress on fat deposition [35], insulin sensitivity [36], and the facilitating role of HSPs in adiposity [37-39]. The most direct evidence supporting our hypothesis comes from studies conducted on pigs. Exposure to low or high ambient temperatures directly impacts heat production and energy balance in pigs [40]. Heat stress causes enhanced adiposity [41] and growth performance through reduced energy and nitrogen use [42]. However, it is noteworthy that novel observations challenge this long-held traditional dogma, linking reduced productive output during heat stress to decreased nutrient intake. It is now clear that the heat stress response profoundly changes postabsorptive carbohydrate, lipid, and protein metabolism independently of decreased feed intake through harmonized alterations in fuel supply and use by various tissues [43]. One might argue whether heat stress in pigs is relevant to the effects of food temperature on energy metabolism in humans, especially when there is no direct evidence to show that ingesting hot food raises body temperature to such an extent. In addition, it may be argued that the association of HSPs with obesity does not automatically infer causation, and it is well known that these systems respond to metabolic stress. Therefore, to test this hypothesis, we prepared a short proposal to conduct a clinical trial, as described in the *Methods* section.

Altogether, these trials might provide preliminary evidence that HSPs contribute to obesity and weight regain. There is evidence that other HSPs, that is, HSP-A12A, regulate adipocyte differentiation and diet-induced obesity through positive feedback regulation with PPARγ (peroxisome proliferator-activated receptor gamma) [44].

To explore the mechanism of the effects of heat stress on porcine adipocytes, Qu et al [35] used an in vitro adipocyte differentiation model to determine the cellular responses that occur during adipocyte differentiation in pigs. Stromovascular cells (preadipocytes) were differentiated for 9 days at a normal (37 °C) or heat stress (41.5 °C) temperature under 5% CO_2_. They measured the expression of a long list of heat stress genes, such as HSP-27, HSP-60, HSP-70, and HSP-90, as well as cellular triglyceride and ATP concentration. Expectedly, heat stress significantly enhanced the expression of HSP genes but surprisingly had no effect on the level of PPARγ, although C/EBPα was significantly induced. Heat stress leads to the enhanced expression of genes involved in fatty acid uptake and cellular triglyceride synthesis. With the progression of differentiation, the total cellular ATP significantly decreased. However, cells under heat stress had significantly greater cellular ATP than those under control temperature. They concluded that heat stress promoted increased adipocyte triglyceride storage, possibly through the upregulation of genes involved in fatty acid uptake and triglyceride synthesis [35]. It was later determined that the induction of phosphoenolpyruvate carboxykinase expression in adipose tissue by heat stress indicates that increased glyceroneogenesis might also be involved in increased fat storage in pigs under heat stress [38].

Although exposure to a higher dietary temperature is a different form of exposure to higher environmental heat in animals, these animal studies [35,40-42] have invaluable lessons that can be translated into a clinical trial. The temperature range of heat stress applied usually lies between 37 °C and 41.5 °C for in vitro studies [35], which can theoretically simulate physiological responses during eating and drinking of hot meals and drinks, and ranges between 12 °C and 29 °C for exposure to ambient temperature studies [40], which is an acceptable temperature difference range (Δ) to study higher or lower dietary temperatures in humans.

Participants with obesity showed increased expression of HSP-60, HSP-72, HSP-90, and GRP-94 levels, specifically influencing BMI and percent body fat [45], whereas insulin resistance resulted in a decrease in intracellular HSP-70 levels. It also appears that weight loss can reduce HSP activation, as demonstrated in a recent trial where acupuncture in combination with dietary restriction was effective in reducing all immunologic factors (anti-HSP antibodies including anti–HSP-27, anti–HSP-60, anti–HSP-65, and anti–HSP-70) and weight loss [46]. Altogether, these findings indirectly support the hypothesis that a network composed of different HSPs (mainly HSP-70 and HSP-90, but including other HSPs, possibly via modulation of insulin sensitivity or resistance) may alter energy balance and contribute to weight gain and loss, which may have important implications in obesity treatment and prevention.

#### Thermic Response

Although the higher temperature of ingested meals and fluids may have some overlap with specific dynamic action (SDA), it should be emphasized that these are 2 different concepts; meal or fluid temperature is a subset of SDA, whereas SDA, or thermic response, is a physiological phenomenon, which comprises the energy expended on all activities of the body after the ingestion of a meal and leads to a rapid postprandial increase in the metabolic rate. SDA is influenced by the size, type, composition, and temperature of the meal as well as body size, body composition, and multiple environmental factors (such as gas concentration and ambient temperature). In humans, a commonly used estimate of the thermic effect is approximately 10% of the caloric intake; however, the average maximum increase in metabolic rate originating from digestion could reach 25% [47].

However, there is indirect evidence that states that meal temperature might influence energy balance, which is not accounted for by SDA. Meal temperature is one of the determining factors of gastric emptying and absorption of food compositions such as carbohydrates in the duodenum [48], which consequently affects heart rate [8] and thereby metabolic needs. In endotherms, including humans, if meals and fluids are ingested at a temperature lower than the core body temperature, extra heat should be generated to elevate the meal temperature to the body temperature. Thus, the cost of food warming is indicated by the SDA response [47]. The contribution of food warming to SDA should vary as a function of meal temperature and mass. In other words, more energy is expended to warm a large, cold meal compared with a smaller, warmer meal [49]. It might be conjectured that the reverse may hold true for warmer meals and fluids and thereby contribute to energy expenditure and thus energy balance.

The results of direct or indirect calorimetry studies can be criticized; these studies are usually short term and do not account for the influence of between-individual differences in dietary temperatures, neither on the day of calorimetry measurements nor the day before. It might be a good practice to advocate the measurement or control of meal temperature during the day of measurement and at least 1 week before indirect calorimetry. A careful appraisal of the methodology of most laboratory studies shows that, at best, participants are asked to refrain from caffeine and alcohol intake and any vigorous physical activity 1 day before the calorimetry study or a standard diet is prescribed [50-54]. It can also be argued that heat acclimation may partially promote thermoregulatory adaptations in people who consume hot foods and fluids. This might be true; however, this has not been experimentally tested. In addition, there might be an interaction between dietary temperature and some ingredients of food composition (for instance, capsinoids) or some dietary behaviors (such as the frequency of consuming ingredients like capsinoids) that might circumvent thermoregulatory pathways.

In a recent meta-analysis, increases in HSP-70 protein expression after heat acclimation were shown to be moderated by the number of heat acclimation days [12]. In other words, there is a strong possibility that between-individual differences in eating behaviors in terms of the temperature of foods and drinks they consume may influence HSP-70 expression and, if HSP-70 expression contributes to energy balance, then this will confound matching protocols in clinical trials, such as treatment-matching, subject-matching, and data-matching protocols.

### Evidence From the Effect of Dietary Temperature on Food and Liquid Intake

Although the current hypothesis is centered on the influence of dietary temperature on HSPs to cause changes in metabolic processes that favor energy storage, a potential confounding effect that may exist is the effect dietary temperature may have on eating behaviors. In an experiment conducted on rats, no interaction was reported between nurture temperature and meal temperature. The food temperature per se had no significant effect on food intake [55]. In contrast, food temperature had an impact on food intake in patients who were hospitalized. The percentage of hospitalized patients who rated the meal temperature as good was higher for those served with isothermal trolleys than those who were not. The amount of food consumed by patients served with isothermal trolleys was significantly greater than that consumed by patients served without the trolleys [56]. Similar results have been obtained in another hospital setting [57]. Furthermore, serving temperature may affect expected satiety and complementary food purchases. Consumers are more likely to choose complementary food items when they consume or intend to consume a food or beverage served cold rather than hot [58], and warm temperature attenuates the preference for savory meals [59]. The dietary temperature may additionally influence executive function and processes associated with obesity, whereas lower executive function acts as a risk factor for increases in specific eating behaviors that play a role in the development of weight management problems [60,61]. Therefore, the dietary temperature may have a complex interaction with total energy consumption, which deserves to be clinically investigated under controlled conditions.

The aforementioned literature justifies the need to conduct a well-controlled clinical trial to test whether higher dietary temperatures interfere with body and cellular energetic states and, thus, influence or confound the results of clinical trials that aim to compare or evaluate dietary interventions.

## Methods

### Overview

To the best of our knowledge, no studies are available regarding the possible contribution of dietary temperatures to the HSP response. We provide preliminary evidence supporting our hypothesis that higher dietary temperatures disproportionally induce activation of both intracellular and extracellular HSPs and that these HSPs influence energy balance and obesity. In this paper, we outline a protocol to test these hypotheses and elucidate the potential roles dietary temperature plays in obesity development.

### A Proposal to Conduct an RCT

A 4-arm randomized design investigating diets served at different temperatures in 5 °C increments (with core body temperature as a reference) in healthy adults would be the first step to elucidate the potential mechanism linking higher meal temperature with energy contribution. The same protocol can be replicated in patients who are overweight and obese or in people with T2DM. In addition, a crossover design can be performed with necessary modifications.

### Participants: Inclusion and Exclusion Criteria

The participants will include 80 healthy (BMI 25-35 kg/m^2^) men and women aged 19-65 years. Entry criteria will include people who are not currently dieting to lose weight and have had no weight loss or gain >2 kg over the past 3 months; not taking any medications (including antifever) that affect energy expenditure or eating; not using tobacco; not pregnant or lactating or planning to become pregnant in the next 12 months; and have no limitations to eating specific foods or to swallowing (eat and drink) a normally estimated range of meal temperatures (37-52 °C). The participants must not have major health problems; known cardiovascular (cardiac, peripheral vascular, and cerebrovascular), pulmonary (chronic obstructive pulmonary disease, interstitial lung disease, and cystic fibrosis), or metabolic (diabetes, thyroid disorders, and renal or liver disease) disease; and severe fever.

### Procedures

The day before the experimental session, the participants will be instructed to avoid alcohol, caffeine, and strenuous exercise and to drink plenty of water for 24 hours. On the day of the experimental session, the participants will be asked to arrive at the laboratory adequately rested and will be provided with boluses of soups (each 100 ml) with a predefined temperature to be taken once (to overcome the problem of temperature dissipation).

The energy content of bolus diets (including the thermoneutral diet and temperature-graded bolus diets) will be matched to each of the participant’s objectively calculated energy demands [62] and will be provided in the following manner: 20% energy for breakfast, 50% for lunch, and 30% for dinner. This procedure will facilitate the administration of boluses with graded temperatures (eg, +5 °C increments) with a fair precision from 37 °C to 52 °C, which is both ethical and within the optimal drinking temperature of 57.8 °C and <85 °C for the maximum temperature used to serve hot drinks such as coffee [63].

After assuring sufficient hydration (urine-specific gravity <1.025) [64], the participants will be instrumented in a thermoneutral room (approximately 25 °C) and then will be entered into a whole-body calorimeter regulated to 35 °C with approximately 20% relative humidity, where they will rest in an upright seated position for a 30-minute habituation period while steady-state measurements will be obtained.

The dietary temperature will be measured using a noncontact thermometer. All drinks and a standardized diet will be served at room temperature to maximize gastric emptying and fluid uptake while minimizing diuresis. It is expected that by using this metered intake to control for intake pacing, ingestion will be completed between 3 minutes and 5 minutes and will control for heat dissipation of foods and fluids as well. To avoid thermal injury while providing a satisfactory sensation to the participants, the optimum temperature for serving hot fluids and meals will be observed [63].

The drinks and meals will be consumed at a speed that is as rapid as the participants can comfortably manage, which is described elsewhere in detail (mean 11 seconds for 200 mL and 38 seconds for 500 mL) [65,66]. The intraesophageal and intragastric temperatures will be monitored using thermocouples, which is a widely used technique [67]. As currently there is no specific dynamic model for real-time monitoring or measuring of temperature changes of digested meals or drinks along the alimentary tract, inner food and drink temperature will be estimated using the inverse heat conduction problem methodology on surface temperature measurements obtained using thermography [68]. For practical and physiological purposes, considering the applied speed of ingestion, the magnitude of temperature changes along the alimentary tract could be considered as negligible [65-67,69,70].

### Measures

All outcome measures will be measured at 0, 6, and 12 weeks. The participants will be asked to follow their assigned diet served at 37 °C, 42 °C, 47 °C, and 52 °C for 12 weeks.

Blood samples will be collected from each participant for analysis after 12-hour fasting, 3 times during the study (at the beginning and 6 and 12 weeks later).

Adipose tissue biopsies, protein isolation, and in vitro cell culture experiments will be performed as described elsewhere [33].

Cellular HSP-70 and HSP-90 levels will be measured to determine the thermic response to feeding, that is, evaluation of the causal relationship among higher dietary temperature, HSP-70/HSP-90 concentrations, and their time profile, as well as measures of obesity, aerobic fitness, resting metabolic rate, and diet-induced thermogenesis.

Food diaries will be analyzed using software to determine energy intake and macronutrient content.

An automated respirometer system plus a whole-body direct calorimeter, as the gold standard, will be used to measure energy expenditure and substrate utilization, aerobic fitness, resting metabolic rate, diet-induced thermogenesis, and markers of protein and fat catabolism to evaluate whether dietary temperature influences metabolism and surrogate end points of obesity in participants, such as weight and waist-hip ratio.

### Duration of Study

The optimum length of the study will be 12 weeks, if feasible, and if not, at least 2 weeks would be necessary.

### Assignment of Interventions

For treatment allocation, randomization (1:1:1:1) and blinding protocol will be used as described previously [71].

### Proposed Outcome Measures

#### Primary Outcome Measures to Be Assessed

*Subset A* denotes the change in BMI, total body fat, body weight, percentage body fat, lean body mass, percentage lean body mass, waist circumference, and waist-hip ratio from baseline to 12 weeks (time frame: 0, 6, 12, and n weeks). The measurements will be performed with light clothing before breakfast and preferably after voiding.

*Subset B* denotes changes in HSP-27, HSP-65, HSP-70, HSP-72, and HSP-90 (in plasma and total leukocytes) and anti–HSP-27, anti-HSP-65, anti-HSP-70, anti-HSP-72, and anti-HSP-90 antibodies from baseline to 12 weeks (or whatever time is feasible, time frame: 0, 6, 12, and n weeks).

*Subset C* denotes changes in motilin, fasting glucose, insulin, C-peptide, 2-hour glucose, and insulin plus homeostasis model assessment–estimated insulin resistance from baseline to 12 weeks (or whatever time is feasible, time frame: 0, 6, 12, and n weeks).

#### Secondary Outcome Measures to Be Assessed

Changes in total cholesterol, triglycerides, low-density lipoprotein, high-density lipoprotein, reactive oxygen species, reactive nitrogen species, and NF-κB from baseline to 12 weeks (or whatever time is feasible, time frame: 0, 6, 12, and n weeks) are to be assessed.

#### Other Outcome Measures to Be Assessed

*Subset A* denotes plasma levels of leptin and adiponectin from baseline to 12 weeks (or whatever time is feasible, time frame: 0, 6, 12, and n weeks).

*Subset B* denotes possible changes in appetite and food intake (using a 3-day dietary recall questionnaire). Immediately before and after each diet, participants will rate hunger, satiety, and prospective food consumption using 100-mm visual analog scales. Gastric motility will be measured using ultrasound imaging systems.

### A Short Proposal to Conduct a Crossover Design

As there are between-individual responses to heat stress [72], there are also interindividual differences in the serum concentration of HSPs [73-75], which may confound statistical analyses when calculating interactions between frequency of drinking or eating hot foods and time elapsed after eating or drinking. To overcome issues with interindividual differences in its broad sense, a crossover design with necessary modifications can be performed. In this case, it will be necessary that participants can eat ad libitum.

A 2-week washout period and thermoneutral diet between conditions will be included, in which participants will eat boluses of soups served at approximately 37 °C. In this case, the order in which participants participate in each treatment will be randomized. The participants will be assigned 4 weeks of each treatment with a 2-week washout period between each of the 4 crossover treatments.

Before starting the second round of the trial, all 3 treatment groups will return to a thermoneutral bolus diet for another 5 weeks (5 weeks of active treatment, 2 weeks of washout period, and 5 weeks of crossover round).

Adipose tissue biopsies and plasma samples will be obtained at 2 time points: before and after dietary treatment diet.

### Statistical Analyses

For statistical analysis, Bayesian adaptive approaches [76] will be applied whenever feasible because this approach is more likely to allocate participants to better-performing arms at each interim analysis.

In addition to standard analyses of RCTs, causal mediation analysis can be performed to determine how much of the weight change is mediated by the increase in HSP levels or appetite or both. Future trials should seek to identify alternative mechanisms with the aim of further refining the intervention.

This seminal clinical trial can provide preliminary data for causal mediation analysis to facilitate the exploration of the causal mechanisms underlying possible energy contributions through higher dietary temperatures. Proper methods will be used for this purpose [77,78].

### Sample Size Calculation

As the choice of type I errors (α, false positive), type II errors (β, false negative), and effect size may be quite arbitrary [79], we suggest calculating the sample size or power using the *R* value (eHSP-70 to iHSP-70 ratio) for a pilot study (alternatively, owing to feasibility, a preliminary study may be conducted on mice or rats).

Furthermore, as providing a priori sample size calculation is misleading and the presentation of CIs—although serving the same purpose—is superior [80], it would be better to use the CI of the *R* value to calculate the sample size based on the feasibility of a priori assumptions about the study results and available resources. In addition, because foods and drinks may be served and consumed at even wider temperature ranges, it would be instrumental to calculate sample sizes based on wider *R* ranges (approximately 0.2-6).

According to Krause et al [30], assuming the ratio *R* = (eHSP-70) / (iHSP-70) = 1 for the controls (resting and unstimulated) and assuming a moderate-hot meal temperature may produce a shift in *R* to up to 5, which is paralleled by an increase in inflammatory markers and stimulation of cell proliferation, *R* values >5 can be considered as an exacerbated proinflammatory response. However, for a conservative calculation, we suggest using *R=*2*.* According to Hunter-Lavin et al [81], heat-shocked cells simultaneously measured HSP-70 at 37 °C (*R*=1.00), 39 °C (*R*=1.45), 42 °C (*R*=0.65), 43 °C (*R*=0.48), and 45 °C (*R*=1.97), this later temperature confirms a proinflammatory response.

Note that at 45 °C (*R*=1.97), there is a turning point compared with that at 43 °C. This means that if a study intends to investigate wider ranges of meal temperature, it is necessary to calculate an array of expected *R* values and then choose the maximum calculated sample size. For an *R* value between 0.22 and 1.1, a sample size of 9 participants is needed to achieve 80% power [1], whereas an *R* value between 1.8 and 6 would require 75 participants to achieve 90% power [2].

Changes in the calculation of the *R* value are suggested to be valid during heat exposure, as *R* values are associated with the heat exposure of peripheral blood mononuclear cells to different temperatures within a physiological range [30]. Precautions that should be considered could include, but are not limited to, correction for noncompliance of the participants, adjustment for multiple comparisons, and innovative study design.

A total of 80 patients will be randomized equally (1:1:1:1) between the 3 active treatments and the thermoneutral group. Overall, 20 participants will be randomly allocated to the thermoneutral diet group (37 °C): 20 at 42 °C, 20 at 49 °C, and 20 at 54 °C. The primary analysis will be intention-to-treat for the whole study cohort.

It is possible that there are multiple roles of HSPs in body weight, weight regain, and composition, probably related to the exposure time during diet ingestion (and also dietary temperature acclimatization during short-term periods, midterm periods, and long-term periods and presence or absence as well as the degree of insulin resistance in people). Thus, conducting a clinical trial for 12 weeks would provide a sufficient time course to investigate this point.

### Further Considerations and Opportunities

Due to HSPs’ intertissue differences [30], it would be helpful to obtain samples of plasma, lymphocytes, central adipose tissue, and muscle (and liver tissue in case of animal study), if feasible, to attempt to find correlations between HSPs and other variables. This study will provide a great opportunity to administer an array of questionnaires to determine the quality of life, anxiety, depression, food frequency, physical activity, etc, and these data in combination with regular examinations will also help to decide whether and when this trial should be continued or stopped. As a preliminary study, it might be proposed to conduct an observational study to retrospectively investigate the mean differences in variables and odds ratios between people who report meals as cold and those who report consuming their meals while it is hot, in terms of serum baseline HSP levels. Subjective dietary temperatures should then be defined as objective temperatures with appropriate measures. This proposed trial can be replicated in healthy people, people with obesity, and people with T2DM (and in long-term patients vs newly diagnosed patients).

### Further Technical Issues That Need to Be Addressed in the Pilot Clinical Trial

One might argue whether the study discriminates between other possible influences of meal temperature on energy balance mentioned in the paper (eg, effect on gastric emptying and diet-induced thermogenesis). Evidently, these issues should also be controlled. Participants can eat ad libitum during the 12 weeks of intervention and are fed only soups for 3 meals a day for 12 weeks and are supposed to stay in the laboratory during the entire experiment. This can confound the assessment of hunger or satiety obtained using the visual analog scale using different meal sizes. If participants receive standardized and isocaloric meals, it can mask a possible effect on appetite as well. A very important issue regarding meal temperature is the experienced pleasantness of the meal, which can influence food intake. This should be assessed as well. We have proposed an intention-to-treat analysis for the pilot study. Such a long intervention probably might result in a significant dropout rate, so it is questionable if sufficient participants will conclude the study.

### Ethical Considerations

As this study involves human participants, the approval by Institutional Review Board (IRB) will need to be obtained before beginning any research activities. This will include an informed consent process, in which participants will be required to provide explicit informed consent for data collection, analysis, and reporting before the inclusion in the study. Such IRB approval will require privacy and confidentiality protection such as the deidentification of participant data, which would be appropriate for this trial. The compensation for participation in research will need to be considered by the specific IRB and research team.

## Results

This trial protocol has not been initiated and funding has not been sought at the time of this publication.

## Discussion

### Principal Findings

Apart from the obvious need to improve weight loss or weight loss maintenance, elucidating the roles that HSP may play in the energy balance is important for future considerations in dietary assessment. The lack of control for dietary temperatures may have produced confounding effects in the data analysis of clinical trials of obesity and medical situations with obesity-related pathology. Dietary assessment tools are widely used in most nutritional and medical studies; therefore, it is critical to have accurate and validated tools. Several meta-analyses and systematic reviews have shown that dietary intake assessment tools have fundamental errors [82-84]; some researchers have proposed that their application in energy balance or obesity and body weight studies must be ceased [85]. It was recently proposed that self-reported energy intake should not be used for the study of energy balance in research on obesity because energy underreporting varies as a function of BMI, and interindividual variability in the underreporting of self-reported energy intake automatically attenuates diet-disease relationships [86]. Furthermore, there is a dispute regarding the validation of dietary assessment tools and the inaccuracy of energy intake measures [87-90]. If dietary temperatures prove to affect energy balance, then future attempts to correct for underreporting and misreporting of diet outcomes cannot avoid taking dietary temperature into account, regardless of whether these tools are kept or abandoned and new tools are developed.

We have presented our hypothesis that dietary temperatures influence energy balance, and we have followed this with a clinical trial that would address this question. The lack of literature regarding possible direct and independent effects of higher dietary temperatures on indexes of obesity warrants a well-designed clinical trial to shed light on the ambiguous role of dietary temperature on daily energy contribution. If meal or drink temperature proves to contribute to energy homeostasis, depending on its contribution and scale, future clinical trials should attempt to adjust this effect when analyzing data. In addition, previous research and established relationships of disease states with dietary patterns, energy, and nutrient intake should be revisited.

This hypothetical proposal is important in several ways. Previous studies [69,70] have only examined the effect of water temperature on gastric emptying and thereby appetite and energy intake; however, they did not consider a potential and direct effect of higher meal or drink temperature through body thermodynamics and HSPs expression. Fujihira et al [69] were the first to investigate how different water temperatures impact gastric motility and energy intake. They showed that consuming 500 mL of water at 2 °C can suppress gastric contractions and ad libitum energy intake compared with consuming 500 mL of water at 37 °C and 60 °C. The subjective appetite perception of hunger in their study tended to be lower after consuming 500 mL of water at 2 °C than at 60 °C. They reported that reduced energy intake after consuming cold water (ie, at 2 °C) is accompanied by a change in gastric contractions. These findings suggest that water temperature may modulate gastric motility to influence energy intake. Indeed, the results of well-conducted experiments confirm that liquid and solid meals at 60 °C accelerated gastric emptying compared with those at 37 °C during the initial 30 minutes [66,70]. However, their findings can be simultaneously criticized, as they have not considered the potential biochemical effect of higher dietary temperatures on cell biology and body thermodynamics. For the first time, we argue that dietary temperature may directly influence energy balance through the induction of HSP-27, HSP-65, HSP-70, HSP-72, and HSP-90 expression.

### Conclusions

Our hypothetical proposal potentially adds new knowledge to the existing literature that food temperature may directly contribute to energy balance and thereby influence weight management. Our proposal has profound and widespread implications. For instance, if dietary temperatures prove to affect energy balance, future clinical trials involving metabolic studies have to take dietary temperature into account. In addition, the results of many published metabolic clinical trials may need to be revisited. The results of this proposal will have deep implications for dietary recommendations, such as the recommended daily energy intake for different age groups and diet planning in different health and disease states. Efficacy studies of nutritional interventions will also need to consider this point. Furthermore, some cultures are accustomed to consuming hot meals and drinks. Comparative studies, particularly those dealing with weight or metabolism, may need to account for biases created by different dietary temperatures. Our hypothesis may also explain the discrepancies in different studies (that did not control for dietary temperature), despite similar methodologies.

The proposed trial does carry limitations. There is no specific dynamic model for real-time monitoring or measuring of temperature changes of digested meals or drinks along the alimentary tract; therefore, we will have to estimate the inner food and drink temperature using the inverse heat conduction problem methodology, which may influence the precision of the results.

## Appendix

Multimedia Appendix 1 Association between indices of body composition and antibody titers to heat shock protein (HSP)–27, HSP-60, HSP-65, HSP-70, and HSP-90.

## Abbreviations

ATP: adenosine triphosphate
HSP: heat shock protein
IRB: Institutional Review Board
PPARγ: peroxisome proliferator-activated receptor gamma
RCT: randomized controlled trial
SDA: specific dynamic action
SGBS: Simpson-Golabi-Behmel syndrome
T2DM: type 2 diabetes mellitus
