# Supplementary material for: Dietary Temperature’s Influence on Energy Balance in Humans: Protocol for a Randomized Controlled Trial and Crossover Design
Source: JMIR Res Protoc. 2023 Mar 3;12:e42846. doi: 10.2196/42846 (PMC10024216; doi:10.2196/42846)
Supplement: Multimedia Appendix 1 [file resprot_v12i1e42846_app1.docx]

**Multimedia Appendix Table .** Association between indices of body composition and antibody titers to heat shock protein (HSP)–27, HSP-60, HSP-65, HSP-70, and HSP-90.

| Reference | Condition^a^ | | | | | | | |
| --- | --- | --- | --- | --- | --- | --- | --- | --- |
|  | Healthy men and women (AU/mL; n=35; BMI 22.3(0.2) kg/m^2^), median (IQR) | | | | Men and women with obesity (AU/mL; n=77; BMI 34.07(0.6) kg/m^2^), median (IQR) | | | |
|  | Anti–HSP-60 antibodies | Anti–HSP-65 antibodies | Anti–HSP-70 antibodies | Anti–HSP-90 antibodies | Anti–HSP-60 antibodies | Anti–HSP-65 antibodies | Anti–HSP-70 antibodies | Anti–HSP-90 antibodies |
| Ghayour-Mobarhan M [24] | 0.22 (0.15-0.30) | 0.30 (0.20-0.53) | 0.20 (0.13-0.29) | — | 0.41 (0.28-0.65)*** | 0.40 (0.25-0.72) * | 0.26 (0.16-0.47) * | — |
|  | Healthy men and women (AU/mL; n=135; BMI 24.3±0.25 kg/m^2^), median (IQR) | | | | Patients with dyslipidemia (AU/Ml; n=237; BMI 29.1±0.33 kg/m^2^), median (IQR) | | | |
|  | Anti–HSP-60 antibodies | Anti–HSP-65 antibodies | Anti–HSP-70 antibodies | Anti–HSP-90 antibodies | Anti–HSP-60 antibodies | Anti–HSP-65 antibodies | Anti–HSP-70 antibodies | Anti–HSP-90 antibodies |
| Ghayour-Mobarhan M [25] | 0.22 (0.16-0.30) | 0.31 (0.22-0.50) | 0.19 (0.13-0.27) | — | 0.27 (0.18-0.37)** | 0.45 (0.28-0.79)*** | 0.22 (0.17-0.30)* | — |
|  | Participants with normal weight (AU/mL; n=50; BMI 25 kg/m^2^), median (IQR) | | | | Participants with obesity (AU/mL; n=100; BMI ≥30 kg/m^2^), median (IQR) | | | |
|  | Anti–HSP-27 antibodies | — | — | — | Anti–HSP-27 antibodies | — | — | — |
| Tavallaie S [22] | 0.18 (0.10-0.23) | — | — | — | 0.34 (0.20-0.39)* | — | — | — |
|  | Healthy, pregnant women (AU/mL; n=127; BMI 26.0 [23.7-28.0] kg/m^2^), median (IQR) | | | | Patients with preeclampsia (AU/mL; n=93; BMI 29.4 [26.3-32.0] kg/m^2^), median (IQR) | | | |
|  | Anti–HSP-60 antibodies | Anti–HSP-65 antibodies | Anti–HSP-70 antibodies | HSP-90 antibodies | Anti–HSP-60 antibodies | Anti–HSP-65 antibodies | Anti–HSP-70 antibodies | HSP-90 antibodies |
| Molvarec A [26] | 34.7 (20.4-55.6) | 9.6 (5.9--15.8) | 229 (149-391) | — | 33.6 (20.9-52.2) | 8.6 (5.4-13.7) | 206 (163-294) | — |

^a^Between-group comparisons were assessed by using the Kruskal-Wallis test as they are nonnormal distribution data; Most of the papers did not report a relationship between HSP and BMI, so a consistent reporting was not possible. Therefore, we had to suffice to citing the anti-HSP antibodies only.

— Data not available

**P*<.05.

***P*<.01.

****P*<.001.
